# PPARγ2 Pro12Ala Polymorphism is Associated in Children With Traits Related to Susceptibility to Type 2 Diabetes

**DOI:** 10.3389/fphar.2021.763853

**Published:** 2021-11-23

**Authors:** Claudia Vales-Villamarín, Olaya de Dios, Iris Pérez-Nadador, Teresa Gavela-Pérez, Leandro Soriano-Guillén, Carmen Garcés

**Affiliations:** ^1^ Lipid Research Laboratory, IIS-Fundación Jiménez Díaz UAM, Madrid, Spain; ^2^ Department of Pediatrics, IIS-Fundación Jiménez Díaz UAM, Madrid, Spain

**Keywords:** PPARγ2 Pro12Ala polymorphism, insulin, HOMA, obesity, body mass index (BMI), children

## Abstract

Peroxisome proliferator-activated receptor gamma (PPARγ) is a ligand-activated nuclear receptor that regulates glucose and lipid metabolism. Pharmacological activators of PPARγ are being used as a treatment of obesity related disorders such as dyslipidaemia and type 2 diabetes, but questions remain open regarding the effects of PPARγ on traits related to the development of type 2 diabetes. In our study, we have analyzed the relationship of the common variant Pro12Ala in the human PPARγ2 gene with the presence of obesity and with insulin, HOMA and lipid profile in a representative sample of 6-to 8-year-old children free from the confounding factors associated with adults. We found that Ala12Ala genotype was significantly more frequent in females with obesity than in those without obesity, with Ala12Ala carriers having significantly higher weight and body mass index (BMI), however the association disappeared when adjusting by leptin concentrations. The Ala12Ala genotype was associated with significantly higher HDL-cholesterol and apoA-I levels in males but not in females, independently of BMI. In a recessive model, in females, leptin levels appeared higher in Ala12Ala carriers. Although no apparent differences were observed in any sex when analyzing insulin levels and HOMA among genotypes without adjusting, lower insulin levels and lower HOMA appeared associated with Ala12Ala carriers when adjusting for BMI and leptin levels. In summary, our data showed that leptin seems to be having an effect on the association between the PPARγ2 Pro12Ala and BMI. Besides, after controlling for BMI and leptin, a protective effect of the Ala12Ala variant of the PPARγ2 Pro12Ala polymorphism on insulin sensitivity is evident already in prepubertal children.

## Introduction

The dysregulation of the homeostatic processes associated with obesity results in metabolic disorders originating atherosclerosis and type 2 diabetes. Treatment of the obesity-related complications related to type 2 diabetes is challenging. Among the genes that influence insulin sensitivity those playing a role in adipose tissue may be considered as good candidates. The peroxisome proliferator-activated receptors (PPARs) are lipid-activated transcription factors that modulate several biological processes that are altered in obesity, including lipid and glucose metabolism and overall energy homeostasis (Gross et al., 2017). Among the PPAR family, PPARγ plays a central role in glucose homeostasis and adipocyte differentiation and has been related with diabetes mellitus (Kwak et al., 2002). In this sense, PPARγ ligands of the antidiabetic thiazolidinediones group are being used as a treatment of obesity comorbidities such as dyslipidaemia and type 2 diabetes (Cariou et al., 2012), but questions have arisen in this regard (Gross et al., 2017) as the development of these pharmacological activators of PPARγ requires a complete knowledge of the PPARγ-regulated control of glucose and lipid metabolism (Cataldi et al., 2021). A deeper knowledge of PPARγ physiological role will contribute to better understand the functionality of these PPARγ agonists on preventing type 2 diabetes.

PPARγ2, being a thiazolidinedione receptor, plays an important role in adipocyte differentiation and gene expression (Spiegelman, 1998). The most common genetic variant in the human PPARγ2 gene is a missense mutation in the exon 2 of the gene that results in a substitution of proline by alanine in codon 12 (named Pro12Ala) (Yen et al., 1997). This variant has been found to modulate the transcriptional activity of the gene (Deeb et al., 1998) and the Ala12 variant was initially associated with lower BMI (Deeb et al., 1998). However, two different meta-analyses showed that this Ala12 variant was significantly associated with higher BMI (Masud et al., 2003; Tönjes et al., 2006). On the other hand, the polymorphism has been extensively studied in relation to type 2 diabetes mellitus (Li et al., 2019). Several studies have associated the Ala12 variant with improved insulin sensitivity and a reduced risk of type 2 diabetes (Altshuler et al., 2000; Hara et al., 2000; Douglas et al., 2001; Ek et al., 2001; Ghoussaini et al., 2005; Radha et al., 2006; Florez et al., 2007), while other have failed to find any association with insulin sensitivity or type 2 diabetes or reported different findings depending if the study is performed in subjects with diabetes or control populations (Mori et al., 1998; Mancini et al., 1999; Ringel et al., 1999; Oh et al., 2000; Rosmond et al., 2003; Martínez-Gómez et al., 2011). Thus, as summarized in the Huge review and meta-analysis, there is an important heterogeneity in the extent of the association among populations (Gouda et al., 2010). This heterogeneity could be attributed to a different effect of PPARγ2 gene among races, gender and age or differences in BMI. In young children, the absence of exposure to many secondary factors associated with adults (smoking, alcohol, pharmacological treatments, etc.) and the lack of influence of sex hormones should permit a better analysis of the influence of these genetic determinants on the variables under study.

In the present work we analyze the relationship of the PPARγ2 Pro12Ala gene polymorphism with the presence of obesity and assess the association of the polymorphism with insulin levels, insulin sensitivity status, estimated using the homeostasis model assessment for insulin resistance (HOMA), and lipid levels in a representative sample of 6-to 8-year-old children.

## Materials and Methods

### Subjects

The sample included 1,254 healthy school children 6–8 years old (633 males/621 females), with an average age of 7.2 years, who participated in a voluntary survey of cardiovascular risk factors in Spain, in whom information on biochemical variables was available. Information on anthropometric variables was available in 1,000 children (499 males and 501 females). More detailed information about the design of the study is available in previous publications (Garcés et al., 2005). The study protocol complied with Helsinki Declaration guidelines and Spanish legal provisions governing clinical research on humans, and was approved by the Clinical Research Ethics Committee of the Instituto de Investigación Sanitaria-Fundación Jiménez Díaz (PIC016-2019 FJD). Parents of all children invited to participate in the study were required to sign a written authorization.

Anthropometric measurements: Measurements were taken with the children lightly dressed and barefoot. Height was measured to the last millimeter using a portable stadiometer and weight was recorded to the nearest 0.1 kg using a standardized electronic digital scale. From these measurements, body mass index (BMI) (weight in kilograms divided by the square of the height in meters: kg/m^2^) was then computed. Children were classified as having obesity if their BMI exceeded the age- and sex-specific cut-off points established for children by Cole et al. (Cole TJ. et al., 2000), a classification that provides an internationally acceptable definition of obesity in children form 2–18 years.

### Biochemical Data

Fasting (12-h) venous blood samples were obtained by venipuncture into Vacutainer tubes. Once centrifuged, the fractions were separated and frozen at –70°C. Plasma cholesterol and triglycerides (TG) were measured enzymatically (Menarini Diagnostics, Italy) with an RA-1000 Autoanalyzer. The coefficients of variation of the methods were 2.06% for cholesterol determinations and 3.42% for triglyceride determinations. HDL-cholesterol (HDL-C) was also measured in the RA-1000 after precipitation of Apo B-containing lipoproteins with phosphotungstic acid and Mg (Roche Diagnostics, Spain). LDL-cholesterol (LDL-C) was calculated according to Friedewald`s formula. Plasma Apo AI and Apo B concentrations were quantified by immunonephelometry (Dade Berhing, Germany). Serum insulin concentrations were measured by RIA using a commercial kit (BI-Insulin IRMA, Bio-Rad, France). Insulin resistance was estimated using the homeostasis model assessment for insulin resistance (HOMA = fasting insulin [μU/ml] × fasting glucose [mmol/l]/22.5). Leptin concentrations were determined by ELISA using a commercially available kit (Leptin CAN-L-4260, Diagnostics Biochem Canada Inc.).

### Genotype Analysis

DNA was isolated from 10-ml EDTA-blood samples by standard procedures. To determine the PPARγ2 Pro12Ala polymorphism (rs1801282), DNA was amplified in a 50 µL reaction volume containing 10 mM deoxynucleotide triphosphates, 50 mM each primer, and 50 mM MgCl. The primers used were: forward, 5′TCT​GGG​AGA​TTC​TCC​TAT​TGG​C 3´; reverse, 5′CTG​GAA​GAC​AAC​TAC​AAG​AG 3´. The thermal cycling conditions were denaturation at 94°C for 5 min and 30 cycles of 94°C for 30 sg, 52°C for 30 s, and 72°C for 30 s. The 154-bp amplified fragment was restricted overnight with the enzyme HhaI and 37°C, and the DNA fragments were resolved by 8% polyacrylamide gel electrophoresis.

### Statistical Analysis

Statistical analyses were carried out using the IBM SPSS software package (Chicago, Illinois, Version 25.0) and GraphPad Prism statistical software (San Diego, California, Version 8). Genotypic and allelic distributions between children with and without obesity were compared using the chi-squared and Fisher´s tests. The normality of the distribution of the variables under study was examined using the Kolmogorov–Smirnov test. Differences in mean anthropometric parameters and biochemical variables between the three genotype groups were tested by the Kruskal–Wallis test. The Mann–Whitney *U* test was used to test for significant association under a recessive model for the Ala allele and to compare the studied variables between sex. Univariate analysis of variance was used to examine the association of the polymorphism with the biochemical variables after adjusting for the cofounder variables.

## Results

Anthropometric and biochemical parameters in males and females in our study are shown in Table 1. The genotype distribution of the Pro12Ala PPARγ2 polymorphism in our cohort was as follows: 84.4% (*n* = 1,058) homozygote carriers of the wild-type genotype coding for proline (Pro12Pro); 14.9% (*n* = 187) heterozygote carriers (Pro12Ala) and 0.7% (*n* = 9) homozygote carriers of the alanine coding genotype (Ala12Ala). The observed genotype frequencies were in agreement with Hardy-Weinberg equilibrium. The prevalence of the Ala12 allele was 8.2%. Of the total of the children included in our study, information on anthropometric variables was available for 1,000 children. Table 2 shows the genotype distribution and allele frequency of the Pro12Ala PPARγ2 polymorphism in males and females classified according to weight category. A significant difference in the prevalence of Ala12Ala genotype was observed between females with and without obesity (6.3 and 0.2%, respectively; Fisher’s test: *p* = 0.003). No differences were observed in males. The analysis of the association of the genotypes with the anthropometric variables in males and females (Table 3) showed similar results. No significant differences between genotypes were observed in males, while in females Ala12Ala carriers showed significantly higher weight and BMI. However, these significant differences between mean weight and BMI among genotypes disappeared after adjusting for leptin.

**TABLE 1:** Anthropometric and biochemical characteristics (mean ± SD) of males and females.

	Males *n* = 633	Females *n* = 621	*P*
Age (years)	7.2 ± 0.6	7.2 ± 0.6	0.452
Weight (kg)	26.9 ± 5.3	26.7 ± 5.5	0.545
BMI (kg/m^2^)	16.9 ± 2.4	17.0 ± 2.5	0.654
TC (mg/dl)	181.9 ± 26.2	183.8 ± 28.4	0.142
TG (mg/dl)	71.2 ± 25.4	73.9 ± 25.9	0.013
HDL-C (mg/dl)	60.2 ± 13.0	58.8 ± 13.1	0.036
LDL-C (mg/dl)	107.4 ± 25.4	110.3 ± 26.7	0.020
Apo AI (mg/dl)	138.3 ± 19.0	135.7 ± 18.9	0.018
Apo B (mg/dl)	68.9 ± 14.1	71.5 ± 14.9	0.000
Glucose (mg/dl)	91.6 ± 8.4	89.3 ± 9.5	0.000
Insulin (μU/mL)	3.4 ± 2.4	3.6 ± 2.7	0.084
HOMA	0.78 ± 0.58	0.81 ± 0.61	0.295
Leptin (ng/ml)	4.7 ± 6.5	8.7 ± 9.0	0.000

*p*-values: Mann-Whitney U test.

**TABLE 2:** PPARγ2 Pro12Ala genotype and allele frequencies (% (N)) according to weight category by sex.

	Males			Females		
	Without obesity	With obesity	*P* ^a^	Without obesity	With obesity	*P* ^a^
Pro12Pro	83.2 (380)	81.0 (34)	ns	84.1 (381)	85.4 (41)	0.003
Pro12Ala	16.0 (73)	19.0 (8)		15.7 (71)	8.3 (4)	
Ala12Ala	0.9 (4)	0.0 (0)		0.2 (1)	6.3 (3)	
Pro	91.14	90.48	ns	91.94	89.58	ns
Ala	8.86	9.52		8.06	10.42	

a *p*-value for comparison of Ala12Ala prevalence between subjects with and without obesity by Fisher’s test.

ns: non-significant.

**TABLE 3:** Anthropometric parameters (mean ± SD) according to the PPARγ2 Pro12Ala genotype by sex.

	Pro12Pro	Pro12Ala	Ala12Ala	*P* ^a^	*P* ^b^
Males (*n* = 499)	*n* = 414	*n* = 81	*n* = 4		
	Non-adjusted				
Weight (kg)	26,8 ± 5.3	27.2 ± 5.6	28.3 ± 3.4	0.514	0.323
BMI (kg/m^2^)	16.9 ± 2.4	17.1 ± 2.5	16.7 ± 1.4	0.850	0.855
	Adjusted by leptin				
Weight (kg)	26.6 ± 0.3	27.7 ± 0.6	28.3 ± 2.5	0.218	0.543
BMI (kg/m^2^)	16.8 ± 0.1	17.3 ± 0.3	17.0 ± 1.0	0.215	0.867
	
Females (*n* = 501)	*n* = 422	*n* = 75	*n* = 4		
	Non-adjusted				
Weight (kg)	26.7 ± 5.4	26.0 ± 5.4	32.5 ± 5.4	0.041	0.036
BMI (kg/m^2^)	17.0 ± 2.6	16.7 ± 2.0	20.4 ± 2.5	0.049	0.018
	Adjusted by leptin				
Weight (kg)	26.8 ± 0.3	25.7 ± 0.6	28.4 ± 2.1	0.143	0.404
BMI (kg/m^2^)	17.1 ± 0.1	16.9 ± 0.3	18.4 ± 0.9	0.265	0.132

a *p*-value for comparison between groups by Kruskal-Wallis test.

b *p*-value for comparison in a recessive model by Mann-Whitney U test.

Tables 4 and 5 show lipid levels, insulin and HOMA, as well as leptin concentrations according to the PPARγ2 Pro12Ala genotypes in males and females respectively. Significant differences (*p* < 0.05) among genotypes were observed for HDL-cholesterol and apo A-I in males. In a recessive model, significantly higher HDL-cholesterol and apo A-I were observed in males homozygous for the 12Ala allele. Significantly higher leptin levels were observed in females’ carriers of the Ala12Ala genotype. After adjusting for BMI, the associations of the polymorphism with the lipid parameters remained significant in males and an association of carriers of the Ala12Ala genotype with lower glucose concentrations emerged, being significant (*p* < 0.05) in females (data shown as supplementary data).

**TABLE 4:** Biochemical variables (mean ± SD) according to the PPARγ2 Pro12Ala genotype in males.

	Pro12Pro N = 535	Pro12Ala N = 93	Ala12Ala N = 5	*P* ^a^	*P* ^b^
TC (mg/dl)	181.5 ± 25.3	181.8 ± 29.4	219.3 ± 38.4	0.064	0.020
TG (mg/dl)	71.2 ± 26.1	70.6 ± 21.3	79.3 ± 23.6	0.668	0.387
HDL-C (mg/dl)	60.5 ± 13.2	57.8 ± 11.8	70.6 ± 9.0	0.016	0.048
LDL-C (mg/dl)	106.8 ± 24.7	109.8 ± 28.5	132.9 ± 32.5	0.095	0.051
Apo AI (mg/dl)	138.4 ± 18.9	136.6 ± 18.9	161.6 ± 14.0	0.015	0.006
Apo B (mg/dl)	68.8 ± 14.1	68.9 ± 14.0	80.1 ± 13.4	0.259	0.100
Glucose (mg/dl)	91.3 ± 8.2	93.2 ± 9.2	95.4 ± 9.3	0.146	0.403
Insulin (μU/mL)	3.3 ± 2.4	3.7 ± 2.3	3.7 ± 2.5	0.176	0.756
HOMA	0.76 ± 0.57	0.87 ± 0.58	0.89 ± 0.67	0.121	0.704
Leptin (ng/ml)	4.8 ± 6.8	3.9 ± 4.1	5.6 ± 8.3	0.566	0.828

a *p*-value for comparison between the three genotype groups using Kruskal-Wallis test.

b *p*-value for comparison under a recessive model for the Ala allele using Mann-Whitney U test.

**TABLE 5:** Biochemical variables (mean ± SD) according to the PPARγ2 Pro12Ala genotype in females.

	Pro12Pro N = 523	Pro12Ala N = 94	Ala12Ala N = 4	*P* ^a^	*P* ^b^
TC (mg/dl)	184.5 ± 28.7	180.2 ± 26.9	177.4 ± 23.8	0.344	0.600
TG (mg/dl)	73.8 ± 26.8	73.4 ± 20.9	88.0 ± 22.8	0.255	0.173
HDL-C (mg/dl)	59.0 ± 13.5	57.8 ± 10.8	60.6 ± 18.0	0.911	0.806
LDL-C (mg/dl)	110.8 ± 26.7	107.7 ± 27.0	99.2 ± 16.9	0.410	0.312
Apo AI (mg/dl)	135.9 ± 19.4	134.8 ± 16.2	136.5 ± 22.8	0.929	0.892
ApoB (mg/dl)	71.7 ± 14.9	71.0 ± 15.3	64.1 ± 8.3	0.459	0.228
Glucose (mg/dl)	89.3 ± 9.7	89.8 ± 7.4	76.2 ± 20.5	0.253	0.194
Insulin (μU/mL)	3.7 ± 2.7	3.6 ± 2.9	3.7 ± 0.9	0.765	0.469
HOMA	0.81 ± 0.61	0.81 ± 0.64	0.69 ± 0.29	0.941	0.901
Leptin (ng/ml)	8.6 ± 9.1	8.3 ± 7.7	20.8 ± 13.3	0.024	0.007

a *p*-value for comparison between the three genotype groups using Kruskal-Wallis test.

b *p*-value for comparison under a recessive model for the Ala allele using Mann-Whitney U test.

**FIGURE 1:**
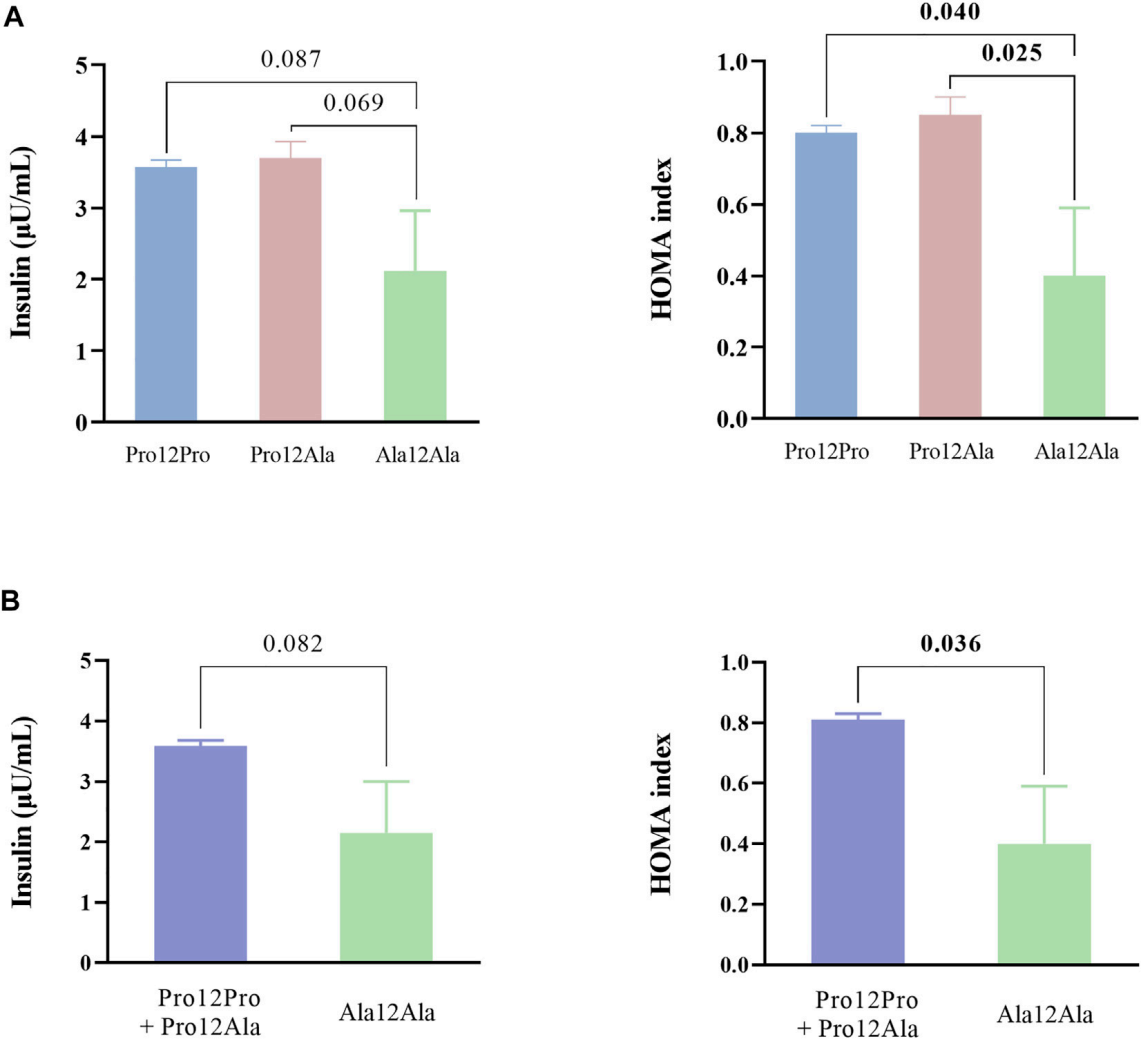
Insulin levels and HOMA values according to the PPARγ2 Pro12Ala polymorphism after adjusting for BMI and leptin levels. **(A)** Differences between the three genotype groups tested using Kruskal–Wallis test. **(B)** Differences under a recessive model for the Ala allele tested using Mann-Whitney U test Data are presented as means with their standard errors. HOMA: homeostasis model assessment of insulin resistance.

Due to the high correlation of insulin and HOMA with BMI and leptin levels, a further analysis of the association of the polymorphism with insulin and HOMA after adjusting for these cofounder factors was performed by univariate analysis of variance. As observed in Figure 1, after adjusting for BMI and leptin levels, homozygote carriers of the alanine coding genotype (Ala12Ala) showed lower insulin levels and HOMA values than heterozygote carriers (Pro12Ala) and homozygote carriers of the genotype coding for proline (Pro12Pro), reaching statistical significance for HOMA (Figure 1A). Significantly lower HOMA values were also observed in Ala12Ala carriers after adjusting by BMI and leptin when analyzing under a recessive model (Figure 1B).

## Discussion

Due to the potential usefulness of activators of PPARγ as a therapeutic option for type 2 diabetes, it is important to understand the relationship of PPARγ with type 2 diabetes related traits. In this sense, the relationship of the PPARγ2 polymorphisms with obesity, insulin sensitivity and lipid profile has been extensively investigated, mainly in adults, with inconsistent results (Deeb et al., 1998; Mori et al., 1998; Mancini et al., 1999; Ringel et al., 1999; Altshuler et al., 2000; Hara et al., 2000; Oh et al., 2000; Douglas et al., 2001; Ek et al., 2001; Masud et al., 2003; Rosmond et al., 2003; Radha et al., 2006; Tönjes et al., 2006; Florez et al., 2007). In this cross-sectional study, the association of Pro12Ala PPARγ2 polymorphism with obesity, plasma lipids, insulin and leptin concentrations, as well as insulin resistance estimated using the homeostasis model assessment for insulin resistance (HOMA) (Matthews et al., 1985) is investigated in a population-based sample of children in whom the effect of several factors such as sex hormones, alcohol consumption, smoking etc. that may contribute to the discrepancies observed in adults can be avoided.

We found that the frequencies of the rare Ala12 allele (8.2%) and the Ala12Ala genotype (0.7%) of this Pro12Ala variant of the PPARγ2 gene in our Spanish children population were similar to that reported for other Spanish (González Sánchez et al., 2002) and Caucasian populations (Beamer et al., 1998; Altshuler et al., 2000; Meirhaeghe et al., 2000; Buzzetti et al., 2005) and higher than in Japanese (Mori et al., 1998) and Korean populations (Oh et al., 2000). When comparing the genotype distribution between children with and without obesity we observed a significantly higher Ala12Ala prevalence in girls with obesity but no significant differences in males. A similar finding has been described in a study including 7–18 years old Italian children (Bordoni et al., 2017), although other study in prepubertal children aged between 4 and 10 years failed to find any direct association between the polymorphism and BMI (Cecil et al., 2005). As reported in two meta-analyses analyzing the association of the polymorphism with BMI, although several studies failed to find any association or reported an inverse association between the 12Ala PPARγ2 variant and obesity, it seems that the Ala12 variant has been consistently associated with higher BMI (Masud et al., 2003; Tönjes et al., 2006). As described by Masud et al. in their meta-analysis, Ala12 homozygotes carriers had significantly higher mean BMI than heterozygotes and Pro12 homozygotes, and authors support the hypothesis that the polymorphism is associated with obesity and the association is consistent with a recessive model (Masud et al., 2003), suggesting that the discrepancies between studies would arise from analyzing together heterozygotes and Ala12 homozygotes. The fact that the association is evident in females and not in males suggests that other factors may be affecting the association of the Pro12Ala PPARγ2 polymorphism with anthropometric variables. PPARγ is a key regulator of adipokines production and secretion. Experimental evidence suggests that PPARγ has a direct effect on leptin gene transcription, down-regulating leptin gene expression (De Vos et al., 1996; Zhang et al., 1996). To our knowledge, studies analyzing the association of the polymorphism with leptin are scarce but, similar to our findings, other studies have found an association of the Ala12 variant with higher leptin levels (Cole SA. et al., 2000; Simón et al., 2002; Becer and Çlrakoǧ, 2017). It can be hypothesized that the Pro12Ala substitution may decrease the suppressing effect of PPARγ on the leptin promoter, affecting leptin concentrations. The higher leptin levels observed in females in our cohort could be contributing to the association between the polymorphism and BMI, as the significant association observed in females disappeared when adjusting by leptin.

One of the main aims of our study was to analyze the association between the Pro12Ala PPARγ2 polymorphism and insulin levels and insulin sensitivity in our children. Some studies in adults have reported an association between the Ala12 variant of PPARγ2 gene and a minor risk of diabetes and insulin resistance (Ek et al., 2001; González Sánchez et al., 2002), although some others failed to find any association (Mancini et al., 1999). Also studies in children have found an association between the polymorphism and insulin sensitivity (Buzzetti et al., 2005; Scaglioni et al., 2006; Dedoussis et al., 2009; Jermendy et al., 2011), although others failed to find this association (Johansson et al., 2009; De Kort and Hokken-Koelega, 2010; Csernus et al., 2015; Stryjecki et al., 2016; Chirita-Emandi et al., 2019). However, most of the studies analyzed data in a dominant model (Stumvoll et al., 2001) and reported significant associations in subjects with obesity (Hara et al., 2000).

In our study in children, we have found no differences in insulin levels and HOMA between genotypes when comparing between genotypes without adjusting for BMI. However, insulin levels and mostly HOMA values appeared lower in Ala12 homozygotes after adjusting for leptin concentrations. According to a recessive model analysis, similar to our results, the minor allele of the Pro12Ala PPARγ2 polymorphism has been associated with insulin levels in healthy men without obesity (Helwig et al., 2007). Carriers of the Ala12Ala genotype showed increased HOMA compared to individuals with Pro12Pro + Ala12Pro genotypes also in diabetic obese children (Dubinina et al., 2014). Li et al. demonstrated significant lower fasting insulin levels and HOMA-IR with the presence of the Ala12 allele in adulthood and a similar trend, although not significant, in childhood (Li et al., 2003). Leptin levels seem to emerge again as a possible confounder factor that may contribute to the discrepancy of results among studies. In our cohort, leptin levels seem to exert an effect on the association between the polymorphism and insulin levels and HOMA. It has been suggested that PPAR ligands reduce obesity-associated comorbidities by acting on fat storage capacity of white adipose tissue and fat burning in brown adipose tissue and/or peripheral tissues (Gross et al., 2017). The fact that when adjusting by leptin this favorable association of the polymorphism with insulin and HOMA emerges supports the role of PPARγ in relationship with insulin resistance already at this age. It has been shown that leptin down-regulates PPARγ mRNA levels in primary human monocyte-derived macrophages (Cabrero et al., 2005). This reduction in PPARγ expression associated with leptin could be masking the beneficial association between PPARγ polymorphism and insulin sensitivity which appears evident when controlling by leptin concentrations.

Regarding lipid parameters, in males, we observed an association of the Pro12Ala PPARγ2 polymorphism with increased levels of HDL-cholesterol and apo A-I that seems to be independent of BMI and leptin levels. An association of the polymorphism with lower triglycerides levels has also been described in children (Muñoz-Yáñez et al., 2016). The role of PPARγ in the treatment of dyslipidaemia has been shown in clinical trials. PPARγ agonists, such as rosiglitazone or pioglitazone, have been associated with an increase in HDL cholesterol levels, but substantial differences in the association with levels of triglycerides and LDL cholesterol have been reported (Soccio et al., 2014). Pioglitazone seems to increase Apo A-I expression, due to activation of PPARα (Zhang et al., 2010), and stimulates reverse cholesterol transport (Cariou et al., 2012). Our data suggest that polymorphisms in the PPARγ genes may contribute to explain differences in the effect of PPARγ agonists on lipid levels due to a different functionality depending on the genotype of the activated PPARγ. In this sense, the review of Khatami et al. supports that PPAR-γ variations should be considered for thiazolidinediones response prediction (Khatami et al., 2019).

As a limitation to our study, we should mention that, due to the design of our study, we are not able to perform the analysis of gene expression levels of each genotype group. Further investigation is needed to clarify this aspect.

In summary, in our study we report that, already in prepubertal children, the Pro12Ala PPARγ2 polymorphism is associated with a positive effect on parameters related to type 2 diabetes mellitus: higher HDL-cholesterol and apo A-I independently of BMI and improved insulin sensitivity after adjusting for BMI and leptin levels.

## Data Availability

The raw data supporting the conclusion of this article will be made available by the authors, without undue reservation.
