# Supplementary material for: PPARγ2 Pro12Ala Polymorphism is Associated in Children With Traits Related to Susceptibility to Type 2 Diabetes
Source: Front Pharmacol. 2021 Nov 23;12:763853. doi: 10.3389/fphar.2021.763853 (PMC8650059; doi:10.3389/fphar.2021.763853)
Supplement: Supplementary file 1 [file DataSheet1.docx]

Supplementary Material

Supplementary Table 1. Biochemical variables (mean (SD)) according to the PPARγ2 Pro12Ala genotype in males after adjusting by BMI.

|  | Pro12Pro  N = 523 | Pro12Ala  N = 94 | Ala12Ala  N = 4 | p* | p# |
| --- | --- | --- | --- | --- | --- |
| TC (mg/dL) | 181.9 (25.1) | 183.2 (30.0) | 206.0 (28.0) | 0.169 | 0.066 |
| LDL-C (mg/dL) | 107.7 (24.3) | 111.3 (29.4) | 121.6 (23.6) | 0.288 | 0.288 |
| ApoB (mg/dL) | 69.3 (14.2) | 69.8 (14.6) | 74.2 (7.7) | 0.804 | 0.535 |
| TG (mg/dL) | 71.5 (22.8) | 71.0 (19.6) | 74.1 (23.7) | 0.914 | 0.772 |
| HDL-C (mg/dL) | 59.8 (12.9) | 57.6 (11.6) | 69.6 (10.1) | 0.112 | 0.117 |
| Apo AI (mg/dL) | 138.1 (18.9) | 137.4 (19.2) | 162.8 (15.9) | 0.033 | 0.010 |
| Glucose (mg/dL) | 91.6 (8.0) | 93.9 (8.8) | 92.2 (6.8) | 0.075 | 0.933 |
| Insulin (μU/mL) | 3.2 (2.15) | 3.6 (2.27) | 2.7 (1.42) | 0.626 | 0.657 |
| HOMA | 0.74 (0.51) | 0.85 (0.57) | 0.61 (0.29) | 0.402 | 0.614 |
| Leptin (ng/mL) | 4.8 (0.30) | 3.2 (0.67) | 2.4 (2.7) | 0.070 | 0.432 |

p*: p-value for comparison between the three genotype groups using Kruskal-Wallis test

p#: p-value for comparison under a recessive model for the Ala allele using Mann-Whitney U test

Supplementary Table 2. Biochemical variables (mean (SD)) according to the PPARγ2 Pro12Ala genotype in females after adjusting by BMI.

|  | Pro12Pro  N = 523 | Pro12Ala  N = 94 | Ala12Ala  N = 4 | p* | p# |
| --- | --- | --- | --- | --- | --- |
| TC (mg/dL) | 185.3 (28.9) | 180.7 (26.9) | 177.4 (23.8) | 0.371 | 0.727 |
| LDL-C (mg/dL) | 111.8 (27.1) | 108.9 (27.5) | 99.2 (16.9) | 0.487 | 0.487 |
| ApoB (mg/dL) | 72.3 (14.9) | 71.3 (15.0) | 64.1 (8.3) | 0.524 | 0.323 |
| TG (mg/dL) | 75.4 (27.1) | 74.6 (20.5) | 88.0 (22.8) | 0.834 | 0.552 |
| HDL-C (mg/dL) | 58.4 (13.3) | 56.8 (10.5) | 60.6 (18.0) | 0.448 | 0.519 |
| Apo AI (mg/dL) | 136.3 (19.5) | 134.7 (16.1) | 136.5 (22.8) | 0.705 | 0.755 |
| Glucose (mg/dL) | 89.7 (9.8) | 89.3 (7.1) | 76.0 (20.5) | 0.005 | 0.001 |
| Insulin (μU/mL) | 3.7 (2.8) | 3.4 (2.1) | 3.7 (0.9) | 0.573 | 0.327 |
| HOMA | 0.82 (0.63) | 0.76 (0.45) | 0.69 (0.29) | 0.342 | 0.153 |
| Leptin (ng/mL) | 8.8 (0.4) | 8.9 (0.9) | 12.3 (3.6) | 0.627 | 0.337 |

p*: p-value for comparison between the three genotype groups using Kruskal-Wallis test

p#: p-value for comparison under a recessive model for the Ala allele using Mann-Whitney U test
